# Genetic assessment of the effect of red yeast (*Sporidiobolus pararoseus*) as a feed additive on mycotoxin toxicity in laying hens

**DOI:** 10.3389/fmicb.2023.1254569

**Published:** 2023-09-06

**Authors:** Shahrbanou Hosseini, Bertram Brenig, Sunattinee Winitchakorn, Chanidapha Kanmanee, Orranee Srinual, Wanaporn Tapingkae, Kesinee Gatphayak

**Affiliations:** ^1^Molecular Biology of Livestock and Molecular Diagnostics, Department of Animal Sciences, University of Goettingen, Göttingen, Germany; ^2^Institute of Veterinary Medicine, University of Goettingen, Göttingen, Germany; ^3^Department of Animal and Aquatic Sciences, Chiang Mai University, Chiang Mai, Thailand; ^4^Functional Feed Innovation Center, Faculty of Agriculture, Chiang Mai University, Chiang Mai, Thailand

**Keywords:** detoxification, feed additive, gene expression, laying hens, mycotoxin, red yeast, RNA sequencing

## Abstract

Toxic fungal species produce hazardous substances known as mycotoxins. Consumption of mycotoxin contaminated feed and food causes a variety of dangerous diseases and can even lead to death of animals and humans, raising global concerns for adverse health effects. To date, several strategies have been developed to counteract with mycotoxin contamination. Red yeast as a novel biological dietary agent is a promising strategy to eliminate mycotoxicity in living organisms. Poultry are most susceptible animals to mycotoxin contamination, as they are fed a mixture of grains and are at higher risk of co-exposure to multiple toxic fungal substances. Therefore, this study investigated the genetic mechanism underlying long-term feeding with red yeast supplementation in interaction with multiple mycotoxins using transcriptome profiling (RNA_Seq) in the liver of laying hens. The results showed a high number of significantly differentially expressed genes in liver of chicken fed with a diet contaminated with mycotoxins, whereas the number of Significantly expressed genes was considerably reduced when the diet was supplemented with red yeast. The expression of genes involved in the phase I (*CYP1A1*, *CYP1A2*) and phase II (*GSTA2, GSTA3*, *MGST1*) detoxification process was downregulated in animals fed with mycotoxins contaminated diet, indicating suppression of the detoxification mechanisms. However, genes involved in antioxidant defense (*GSTO1*), apoptosis process (*DUSP8*), and tumor suppressor (*KIAA1324, FBXO47, NME6*) were upregulated in mycotoxins-exposed animals, suggesting activation of the antioxidant defense in response to mycotoxicity. Similarly, none of the detoxification genes were upregulated in hens fed with red yeast supplemented diet. However, neither genes involved in antioxidant defense nor tumor suppressor genes were expressed in the animals exposed to the red yeast supplemented feed, suggesting decreases the adsorption of biologically active mycotoxins in the liver of laying hens. We conclude that red yeast can act as a mycotoxin binder to decrease the adsorption of mycotoxins in the liver of laying hens and can be used as an effective strategy in the poultry feed industry to eliminate the adverse effects of mycotoxins for animals and increase food safety for human consumers.

## Introduction

Mycotoxins are toxic secondary metabolites produced by filamentous fungi that contaminate agricultural products before harvest in the field or under post-harvest conditions during storage (Brown et al., 2021). Improper storage of crops and grains in high humidity along with high ambient temperature and inappropriate drying methods play an important role in the development of fungi and the production of toxins (Kamle et al., 2022). Toxigenic fungi synthesize different types of mycotoxins, of which aflatoxin B1 (AFB1), ochratoxin A (OTA), zearalenone (ZEA), T-2 toxin (T-2), deoxynivalenol (DON), and fumonisin B1 (FB1) are the important mycotoxins in agricultural products and foodstuff (Agriopoulou et al., 2020; El-Sayed et al., 2020; Srinual et al., 2022). Feed and food contaminated with toxinogenic fungi can cause disease and death in animals and humans, raising global concerns for food safety and health. The most common harmful effects caused by mycotoxins are carcinogenicity, hepatotoxicity, growth and reproduction toxicity, immunotoxicity, neurotoxicity, and mutagenicity (Luo et al., 2021). AFB1 is the most potent carcinogenic toxin among all mycotoxins, affecting mainly the liver (Agriopoulou et al., 2020; Yan et al., 2020). Mutagenicity is another important effect of AFB1 on DNA structure. Its effect is reinforced, when co-occurring with OTA (Mannaa and Kim, 2017), a harmful mycotoxin that damages kidneys and liver by peroxidation of polyunsaturated fatty acids (Kövesi et al., 2019; El-Sayed et al., 2020). ZEA, a product of the toxinogenic fungi of the genus *Fusarium,* is an estrogenic mycotoxin that alters hormonal balance and causes reproductive disorders (Tiemann et al., 2003; Wang et al., 2012; El-Sayed et al., 2020). ZEA is also metabolized in the liver and exerts hepatotoxic, immunotoxic, carcinogenic, and nephrotoxic effects (Chatopadhyay et al., 2012; El-Sayed et al., 2020). Trichothecenes are a large family of mycotoxins, of which types A (e.g., T-2) and B (e.g., DON) are highly toxic and globally widespread (Agriopoulou et al., 2020; El-Sayed et al., 2020). T-2 toxin leads to an increase in lipid peroxidation and a decrease in the activity of glutathione redox systemin the liver (Mézes et al., 1999; Nakade et al., 2018). However, DON causes cytotoxicity that leads to immunosuppression and apoptosis (Awad et al., 2014; Yang et al., 2017; Nakade et al., 2018). Fumonisins (FB) are mycotoxins fatal in animals that cause liver cancer and damage the kidneys (Voss et al., 2001; Agriopoulou et al., 2020). Among the FB analogues, FB1 is the most abundant and dangerous toxin (El-Sayed et al., 2020) that accumulate in the liver of avian species (Laurain et al., 2021).

Feed and food can be contaminated either with a single or simultaneously with multiple toxic fungal substances that can interact in a synergistic or antagonistic manner. Consumption of combined mycotoxins with synergistic effects can increase the risk of adverse health effects in animals and humans (Speijers and Speijers, 2004). Feeding animals with mycotoxin contaminated feeds leads to reduction of feed intake and efficiency, weight gain, and reproductive performance. Furthermore, susceptibility to infectious diseases, impaired immunity, and a higher rate of mortality increased in animals fed with mycotoxin contaminated diet (Eshetu et al., 2016; Santos Pereira et al., 2019; Popescu et al., 2022; Srinual et al., 2022). Harmful fungi are the major cause of feed contamination in poultry (Srinual et al., 2022), which can affect various organs such as liver and kidneys and can impair the immune and nervous systems (Murugesan et al., 2015), resulting in animal health damage and significant economic losses in the poultry industry and posing a safety risk to human consumers (Ochieng et al., 2021). The degree of resistance or susceptibility to different mycotoxins varies considerably in poultry species (Kulcsár et al., 2021). For example, the toxic effect of T-2 is stronger than DON (Dänicke et al., 2001; Dänicke, 2002). Among the vulnerable animals to AFB1, poultry species possess a wide variety of adverse effects in response to AFB1 toxicity (Reed et al., 2019), whereas they are relatively tolerant to the FB1 compared to other animals (Bermudez et al., 1995). The negative impact of feed contaminated with more than one mycotoxin on productivity and health of broilers and laying hens has been often observed in previous studies (Franco et al., 2019; Emmanuel et al., 2020; Ochieng et al., 2021).

So far, various strategies have been developed to control mycotoxin contamination. They are classified into pre-harvest strategies to prevent mycotoxin production and post-harvest strategies to detoxify contaminated feed (Agriopoulou et al., 2020; Wang et al., 2022). The pre-harvest strategies range from good agricultural practices to the use of biological control agents to prevent toxin production (Luo et al., 2018; Agriopoulou et al., 2020; Caceres et al., 2020). However, once mycotoxins are produced, the detoxification of contaminated agricultural products is a major problem that should be addressed through post-harvest strategies (Agriopoulou et al., 2020). Detoxification methods comprise various chemical, physical, and biological treatments, where the biological agents have proven to be more effective, specific, and environmentally friendly (Agriopoulou et al., 2020; Ndiaye et al., 2022). Among biological control agents, yeast is of particular interest and a promising detoxification strategy for the poultry feed industry, being able to significantly degrade mycotoxins by adsorption through the cell wall (Agriopoulou et al., 2020). Red yeast (*Sporidiobolus pararoseus*), a novel yeast used as a mycotoxin binder, acts as a prebiotic with antioxidant properties and possess high nutritional value for improving production traits in poultry species (Tapingkae et al., 2018; Kanmanee et al., 2022; Srinual et al., 2022).

Despite great efforts to control mycotoxin production, contamination of agricultural products with toxic fungi are still a prevalent problem (Kępińska-Pacelik and Biel, 2021), heightening global concerns about adverse effects on animal and human health. Understanding mycotoxin-induced toxicities at cellular level and the genetic mechanism controlling the expression of genes and relevant enzymes involved in metabolic and detoxification processes plays a crucial role in determining the toxic response in animal species (Neal, 1995). Cells produce many detoxification enzymes in response to mycotoxicity to eliminate cytotoxic xenobiotics. The cellular detoxification mechanism consists of three successive phases (Someya and Kim, 2021). In phase I (functionalization reactions), the cytochrome P450 superfamily of detoxification enzymes (CYP450) oxygenates xenobiotics to form a reactive site on the toxic compounds or primarily metabolize toxins; this occurs mainly in the liver. In phase II (conjugation reactions), the reactive site can conjugate with endogenous hydrophilic substances after the xenobiotics have become hydrophilic in phase I, or with less hydrophobic molecules to the hydrophobic xenobiotics, which are then removed from the cell by the transmembrane transporters in phase III (Hodges and Minich, 2015; Someya and Kim, 2021).

Since poultry are fed with a mixture of various cereal grains and oilseed meals (Babatunde et al., 2021), a study of co-exposure to a combination of different mycotoxins is required to assess the increased risk of detrimental health effects in this species. However, the genetic mechanism and genes involved in xenobiotic detoxification metabolism in response to the simultaneous occurrence of multiple mycotoxins and their interactions with organic binders remains to be elucidated. Therefore, the main objective of this study was to investigate the genetic mechanism underlying feeding with red yeast supplementation in interaction with multiple mycotoxins in the liver of laying hens, the central organ for xenobiotic detoxification metabolism (Ponnazhagan et al., 2021), using transcriptome profiling to gain more insights for the development of an effective approach to eliminate the adverse effects of mycotoxins in poultry species and increase food safety to avoid health concerns for human consumers.

## Materials and methods

### Animal husbandry and feeding regimes

For this experiment, a total number of 288 Hy-Line brown laying hens were transferred to the Faculty of Agriculture, Chiang Mai University, Thailand. The animals were divided into 96 cages (1 m × 1 m) with 3 birds per cage and were kept at a temperature of 25 ± 2°C and a light/dark program of 16 h/8 h during the experimental period. All birds were fed with four different diets at 23 weeks of age for a duration of 9 weeks: control diet (CON), CON diet with red yeast supplementation 1.0 g/kg (RY1.0), CON diet contaminated with 100 μg/kg mycotoxins (MT100), and CON diet with a combination of RY1.0 and MT100 (RY1.0 + MT100). The trial was conducted in a randomized design with 24 replications per experimental group. The control diet used as the basal diet in this study consisted of a mixture of different cereal grains and oilseed meals (commercial diet). The ingredients and nutrient values of the control diet were given by Kanmanee et al. (2022). In the feeding trial of this study, red yeast (1.0 g/kg) was added to the control diet as a feed supplement and a liquid medium of mixed mycotoxin solutions (100 μg/kg) was sprayed on the control diet as a feed contaminant (Srinual et al., 2022).

Red yeast was cultivated in a yeast malt extract medium containing yeast extract (4 g/L), malt extract (10 g/L), and glucose (4 g/L), with the initial pH adjusted to 6.0. This medium was sterilized at 121°C for 15 min and transferred to the 5-L, 30-L, and 300-L bioreactors (BE Marubishi Co., Ltd., Pathum Thani, Thailand) after cooling. The cultivation was conducted at 24°C for 3 days. After cultivation, the medium containing red yeast cells was stored at 4°C for 14 days to allow the autolysis and precipitation of the red yeast cells. The supernatant was discarded and the settled red yeast cells were collected and spray-dried to obtain spray-dried red yeast cells. The procedure and conditions for producing of red yeast are described in detail in Tapingkae et al. (2022).

To provide the mycotoxin contaminated feed, five different mycotoxins, including AFB1, T-2, OTA, ZEA, and DON from the company R-Biopharm, Darmstadt, Germany (Trilogy Dried Standard No. TS-104, TS-314, TS-503, TS-401, and TS-310, respectively) were used in this experiment. For this purpose, the control diet was contaminated with a high concentration of mycotoxin of about 100 μg/kg feed per type of mycotoxin as described by Srinual et al. (2022). Mycotoxins concentration in feed was measured before feeding to animals using Liquid Chromatography with tandem mass spectrometry (LC–MS/MS technique) according to Srinual et al. (2022). Mycotoxins in diet were measured in each of the experimental group in three replicates to determine the average mycotoxin residues. The levels of mycotoxins contamination are shown in Table 1. In addition, the adsorption capacity of red yeast at mycotoxin contamination level of 100 μg/kg feed was examined using the *in vitro* gastrointestinal poultry model, which demonstrated that red yeast can adsorb mycotoxins such as aflatoxin B1, zearalenone, deoxynivalenol, T-2 toxin, and ochratoxin A more than 50% (Tapingkae et al., 2022). The contaminated feed was left overnight at room temperature to allow the solvent to evaporate before it was fed to the animals. The toxicity level used in this experiment was in accordance with the European Commission recommendation for maximum toxicity levels of deoxynivalenol, zearalenone, and ochratoxin A (2006/576/EC), T-2 toxin (2013/165/EU), and the maximum permitted level of aflatoxin B1 (574/2011/EC) for poultry.

**Table 1:** The levels of mycotoxins in the experimental diet at 100 μg/kg (100% MT).

Mycotoxins	Mean (μg/kg) ± SD
Aflatoxin B1	99.98 ± 0.15
Zearalenone	99.96 ± 1.00
Deoxynivalenol	99.98 ± 0.17
T-2 toxin	99.97 ± 0.35
Ochratoxin A	99.73 ± 0.62

### Tissue sample collection, RNA isolation and sequencing

Liver tissue from four animals in each experimental group was collected after the feeding treatment at 32 weeks of age. For this purpose, the liver tissue samples were carefully dissected after animal slaughter, snap-frozen and stored at −70°C for further laboratory analysis. To this end, a total number of 16 tissue samples were used for transcriptome analysis in this study. For RNA sequencing (RNA_Seq), total RNA was isolated from all samples using phenol-chloroform + RNeasy mini kit (QIAGEN, Germany) according to the manufacturer’s protocol. The RNA quality and quantity were measured using a Qubit 2.0 Fluorometer (Life Technologies, Thermo Fisher Scientific) and RNA Screen Tape on an Agilent Bioanalyzer 2,100 (Agilent, Santa Clara, USA). The RNA libraries preparation was performed using the NEBNext® Ultra ™II RNA Library Prep Kit for Illumina® and sequenced on an Illumina NovaSeq6000 Platform, aiming PE150 reads per sample.

### RNA read alignment and read counts

The standard bioinformatics pipeline was used for the analysis of the RNA_Seq data in this study. The raw sequencing reads, which were stored in FASTQ format files, were first assessed for quality control. Low quality bases, low quality reads, and Illumina adaptors were removed from the raw data to avoid negative effects on the quality of the downstream analysis. The clean reads were then mapped to the chicken reference genome version GCA_000002315.2, downloaded from the Ensemble website (Flicek et al., 2014), using the HISAT2 version 2.0.5 (Kim et al., 2015, 2019), resulting in an average mapping success rate of 94.4%. Finally, the number of reads mapped for each gene was counted across all samples for downstream analysis using FeatureCounts version 1.5.0-p3 (Liao et al., 2014).

### Differential gene expression and functional annotation analyses

Differential gene expression analysis comparing two conditions (different feeding diets versus control diet) was performed using the DESeq2 R package version 20.0 (Love et al., 2014) in different experimental groups with four biological replications per group. To assess the effect of feeding with different diets on gene expression in the liver of laying hens, we compared RY1.0 vs. CON, MT100 vs. CON, and RY1.0 + MT100 vs. CON. For this analysis, the normalized read counts of all samples from each experimental group were used to determine the differential gene expression by applying the generalized linear model (GLM) underlying a negative binomial distribution. The resulting *p*-values were adjusted for multiple testing correction using the Benjamini and Hochberg approach (Benjamini and Hochberg, 1995), where the expression differences between the compared groups were considered to be statistically significant at *p*_adj_ < 0.05. To further investigate the target genes involved in the xenobiotic detoxification metabolism process in the liver of laying hens, we generated a list of candidate genes collected from the literature and the NCBI gene database for chicken. To this end, a total number of 306 genes, which play an important role in the detoxification metabolism process in response to exposure to mycotoxins in poultry, was investigated as selected candidate genes in this study (Supplementary Table 1, S1).

The functional enrichment analysis was performed using differentially expressed genes (DEGs) to gain more insights into their biological function. The clusterProfiler R package version 3.8.1 (Yu et al., 2012) was utilized for gene ontology (GO) analysis using GO database (The Gene Ontology Consortium, 2015) and pathway analysis using Kyoto Encyclopedia of Genes and Genomes (KEGG) database (Kanehisa et al., 2012). The enriched GO terms and pathways with *p*_adj_ < 0.05 were considered to be statistically significant.

## Results

### Differentially expressed genes in chicken fed with red yeast and mycotoxins and their interaction

**Figure 1:**
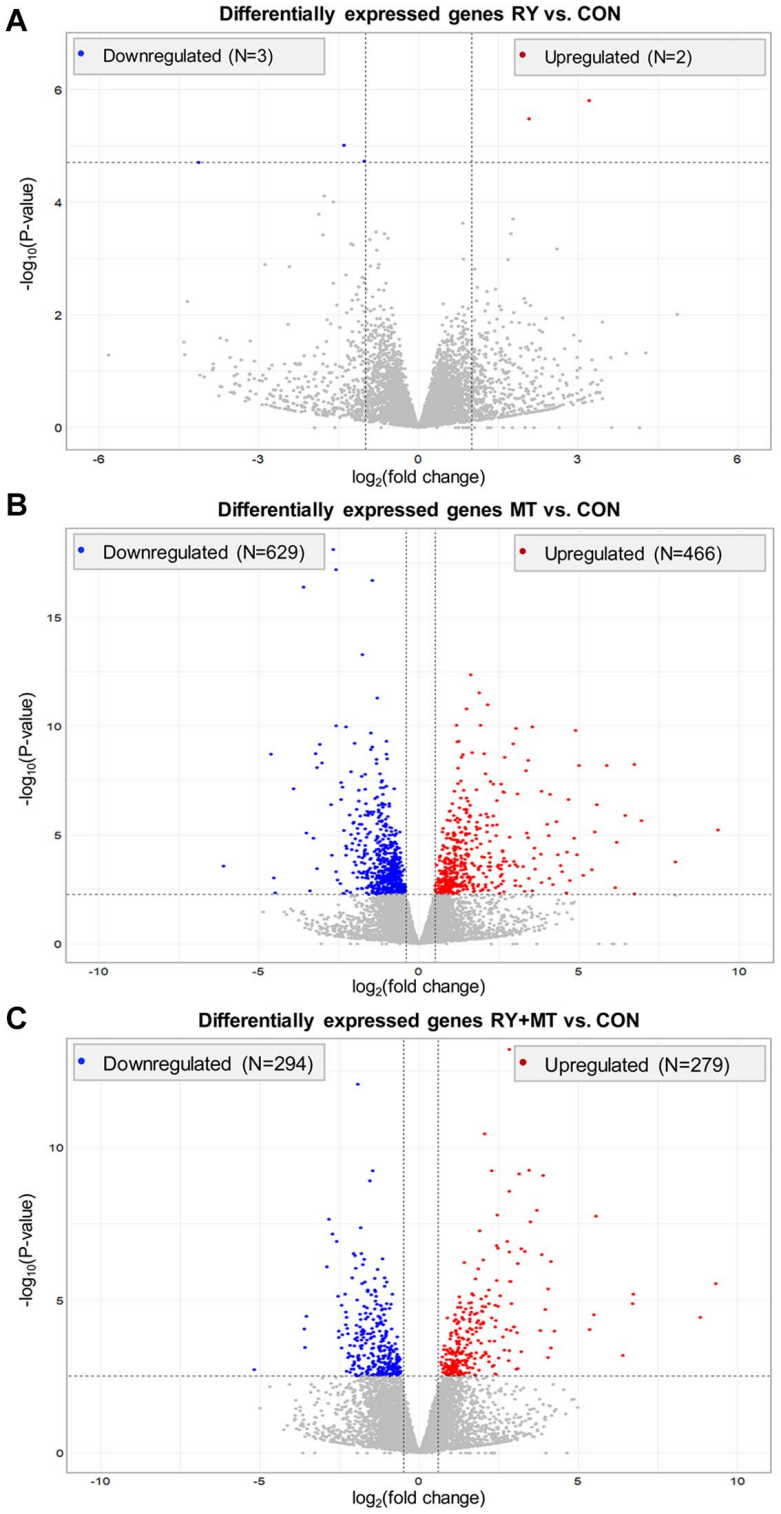
Overall distribution of differentially expressed genes (DEGs) in the liver of laying hens. The volcano plots illustrate significant DEGs in different experimental groups: **(A)** red yeast versus control (RY vs. CON), **(B)** mycotoxins versus control (MT vs. CON), **(C)** red yeast + mycotoxins versus control (RY + MT vs. CON). Each dot in the plots represent a gene with its corresponding log_2_ (fold change) on the *x*-axis and -log_10_ *p*-value on the *y*-axis. The significant expression differences are shown at the significant threshold (*p*_adj_ < 0.05).

To quantify the genetic response to mycotoxin toxicity and red yeast as a biological toxin binder in laying hens, different dietary regimens were applied during the early stage of the laying period. In this comparative study, the liver transcriptome profiles of animals fed with several diets were analyzed in comparison to the control diet in different experimental groups: feed supplemented with red yeast (RY1.0) versus control (RY vs. CON), feed contaminated with mycotoxins (MT100) versus control (MT vs. CON), feed with red yeast supplementation and mycotoxins contamination versus control (RY + MT vs. CON). In this analysis, a total number of 24,356 transcripts were read in the RNA_Seq expression profiles with an average mapping efficiency of 94.4% to the reference genome in the liver of experimental animals (FPKM>1, Fragments Per Kilobase of transcript sequence per Million base pairs sequenced mapped). As expected, the results showed a low number of significantly differentially expressed genes (DEGs, *p*_adj_ < 0.05) in RY vs. CON group (*N* = 8 genes, Figure 1A). However, a high number of DEGs was detected in the MT vs. CON group, where 466 genes were upregulated and 629 genes were downregulated (Figure 1B). This number of DEGs was considerably reduced to 279 upregulated and 294 downregulated genes, when RY was supplemented to the diet in RY + MT vs. CON group (Figure 1C).

### Dietary interaction between mycotoxins-exposed and red yeast + mycotoxins groups

**Figure 2:**
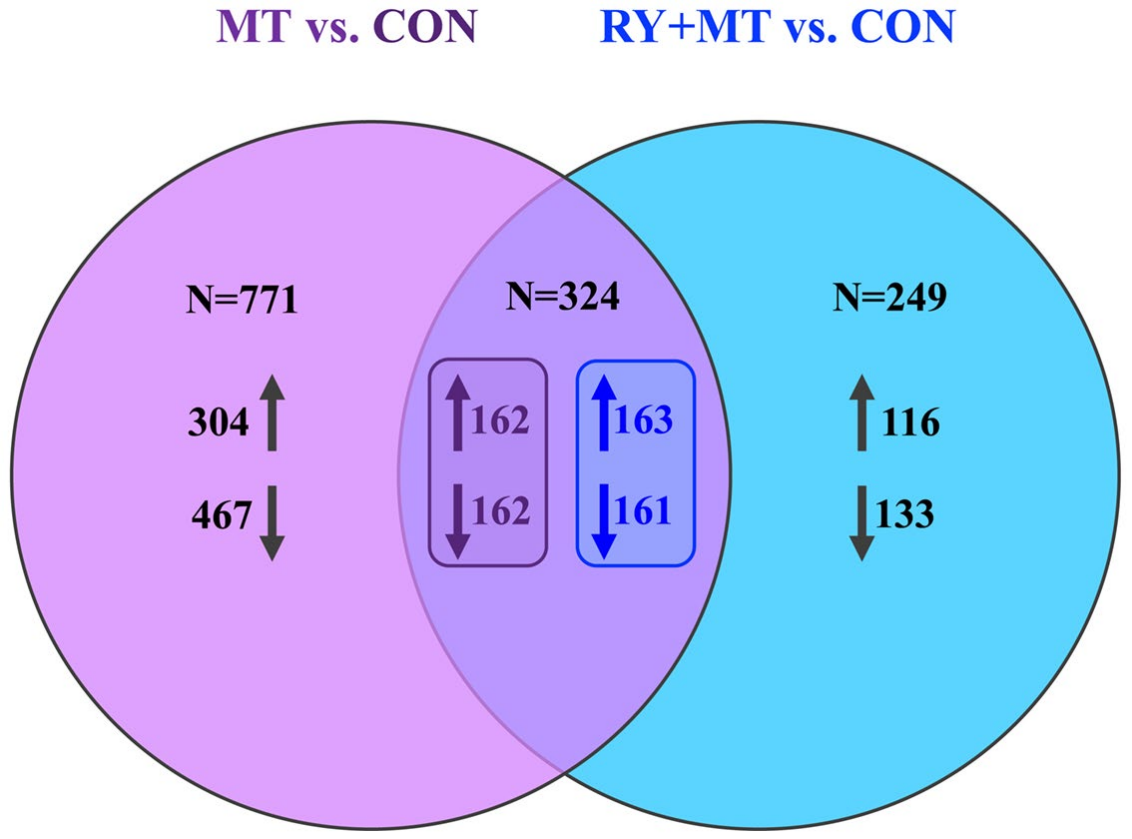
Venn diagram of significantly differentially expressed genes. The Venn diagram shows the number of uniquely expressed genes (upregulated↑ and downregulated↓) comparing mycotoxins versus control (MT vs. CON) and red yeast + mycotoxins versus control (RY + MT vs. CON) groups, with the overlapping region illustrating the number of genes co-expressed in both experimental groups.

A further investigation of the interaction between the MT vs. CON and RY + MT vs. CON groups to determine the beneficial effect of red yeast as a mycotoxin binder on DGEs is shown in Figure 2. The Venn diagram of significant DEGs in the mycotoxins-exposed and red yeast + mycotoxins groups revealed a number of genes that are unequally expressed within each group, where the expressed genes in the RY + MT vs. CON group (*N* = 249) were significantly lower than those in the MT vs. CON group (*N* = 771). The overlapping region indicated the number of co-expressed genes in both experimental groups (*N* = 324), with only a slight difference between the number of upregulated and downregulated genes under the two different feeding regimes.

### Top significantly differentially expressed genes in different experimental groups

**Figure 3:**
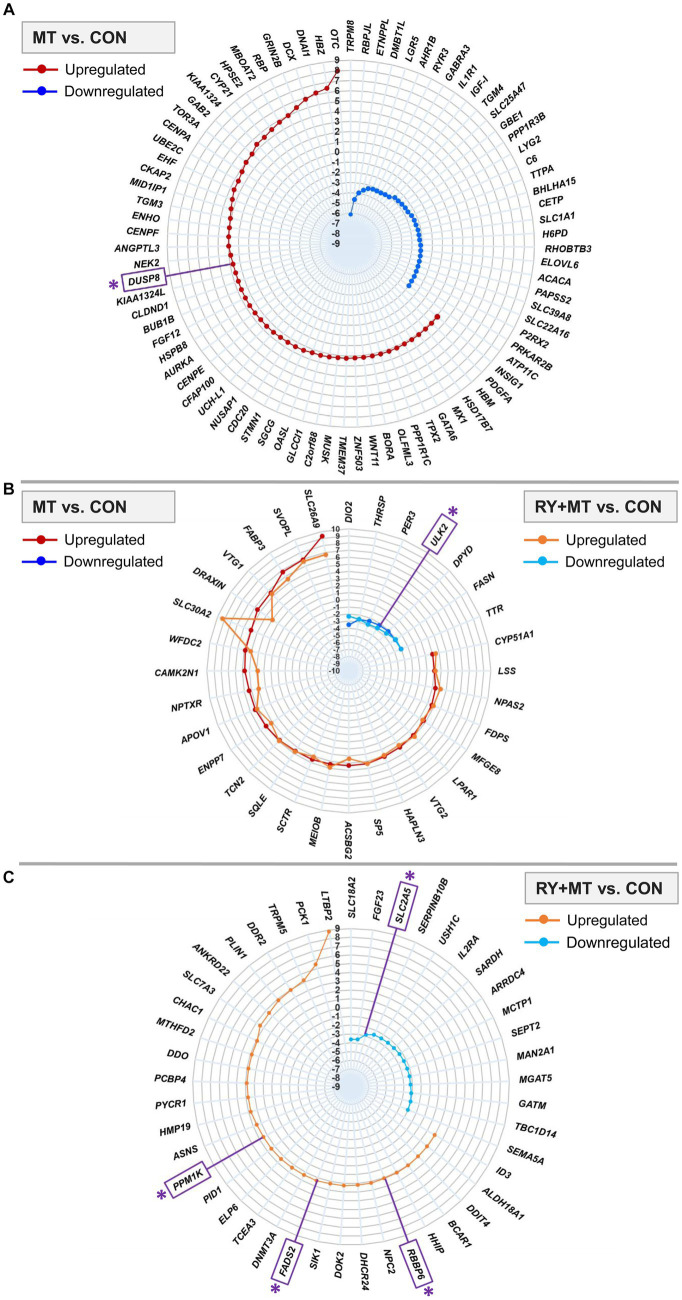
Top significantly differentially expressed genes (DEGs). The radar plots represent the top significantly upregulated and downregulated genes (*p*_adj_ < 0.01, −2 < log_2_ fold change >2) in different experimental groups: **(A)** mycotoxins versus control (MT vs. CON), **(B)** in both mycotoxins versus control (MT vs. CON) and red yeast + mycotoxins versus control (RY + MT vs. CON) groups (common genes), **(C)** red yeast + mycotoxins versus control (RY + MT vs. CON). The identified candidate genes expressed at the top significant level are marked with an asterisk (*) in the figure.

We further focused on identifying the top significant DEGs (*p*_adj_ < 0.01, log_2_ fold change>2) in mycotoxins-exposed and red yeast + mycotoxins groups compared to the control diet (Figure 3). The results showed that 83 genes were significantly differentially expressed in MT vs. CON, in which 53 genes were upregulated and 30 genes were downregulated (Figure 3A). A number of 32 DEGs was identified in both feeding groups (common genes), in which 25 genes were upregulated and 7 genes were downregulated (Figure 3B). The number of identified top significant DEGs in MT vs. CON was reduced to 45 genes in RY + MT vs. CON, with 30 upregulated and 25 downregulated (Figure 3C).

Among the top significant genes in the MT vs. CON group, *OTC*, a gene involved in detoxification of ammonia into non-toxic urea (Caldovic et al., 2015), showed the highest expression level in response to mycotoxin exposure. The upregulation of an apoptosis gene, *DUSP8,* a candidate gene in this study (see M&M), was detected in the MT vs. CON group. Interestingly, in response to mycotoxin-induced toxicity, a tumor suppressor gene *KIAA1324* was upregulated in the same comparison group. By contrast, the expression of *IL1R1*, an important gene involved in many cytokine-induced immune and inflammatory responses, was considerably downregulated in the mycotoxins-exposed group compared to the control animals (Figure 3A).

The genes expressed in both MT vs. CON and RY + MT vs. CON experimental groups (common genes), showed downregulation of a candidate gene [*ULK2*, an apoptosis gene, Liu et al. (2020)] in both groups. However, a pronounced difference in the expression level of common genes was observed for *SLC30A2*, *SLC26A9*, and *DRAXIN*, all of which were upregulated in the mycotoxins-exposed as well as in red yeast + mycotoxins groups (Figure 3B).

In the top significant DEGs in RY + MT vs. CON group, some candidate genes were identified, in which *PPM1K* (essential for cell survival and development), *FADS2* [functional gene in fatty acid metabolism (Liu et al., 2020)], and *RBBP6* [essential for protein ubiquitination process to prevent DNA damage (Dietrich et al., 2012)] were upregulated, and *SLC2A5* [glucose and fructose transporter in cell (Dietrich et al., 2012)] was downregulated (Figure 3C).

### Candidate genes associated with mycotoxins toxicity in the liver of chicken

**Figure 4:**
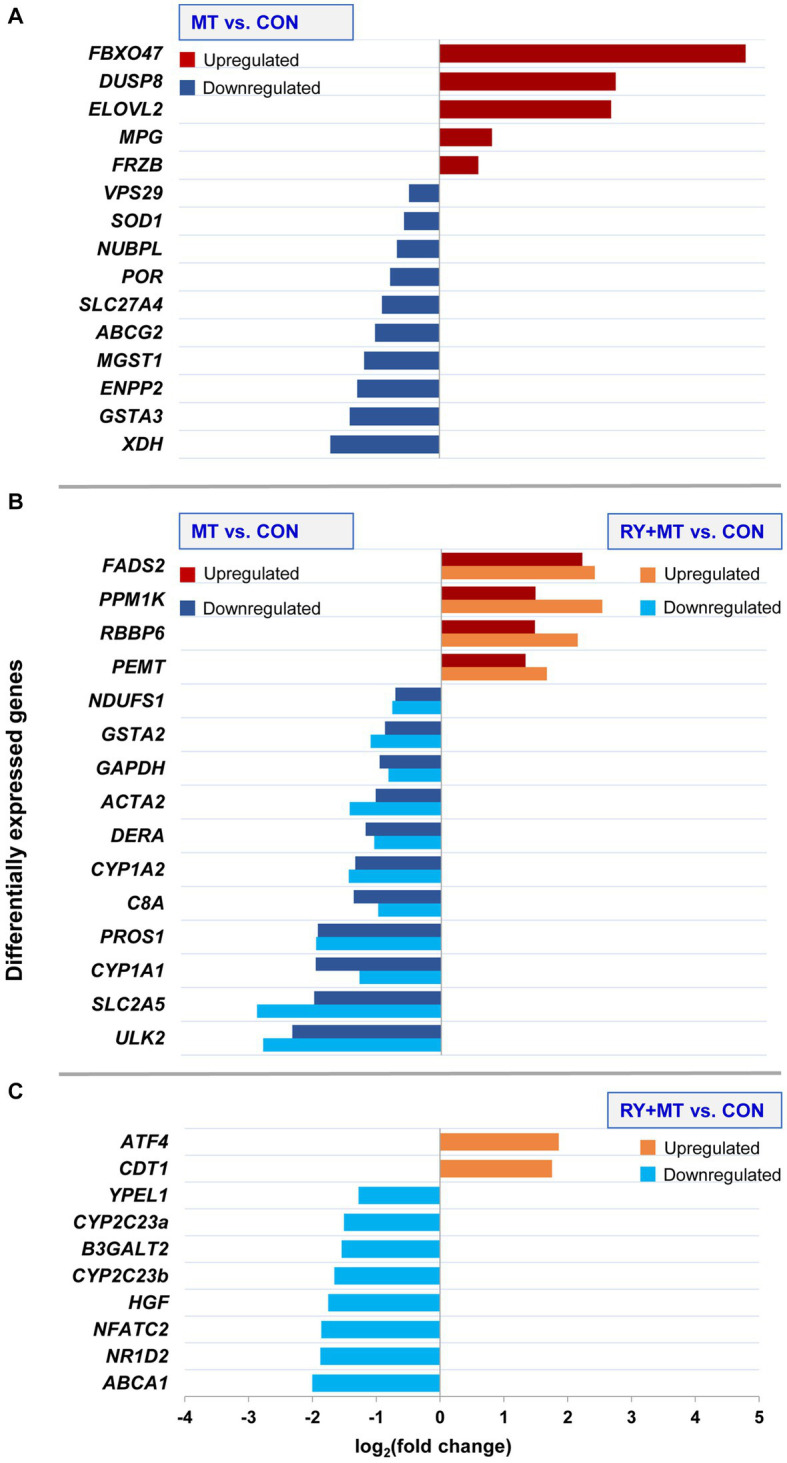
Differential expression of candidate genes. The bar charts illustrate upregulation and downregulation of candidate genes involved in mycotoxin toxicity at the significant threshold (*p*_adj_ < 0.05) in different experimental groups: **(A)** uniquely expressed candidate genes in mycotoxins versus control (MT vs. CON), **(B)** common candidate genes expressed in both mycotoxins versus control (MT vs. CON) and in red yeast + mycotoxins versus control (RY + MT vs. CON), **(C)** uniquely expressed candidate genes in red yeast + mycotoxins versus control (RY + MT vs. CON). The x-axis represents the differences in mean fold change (log_2_) per gene.

The differential expression of candidate genes involved in mycotoxin toxicity in the liver of laying hens fed with different feeding regimes is illustrated in Figure 4. The significant level of up- and down-regulation (*p*_adj_ < 0.05) of a set of candidate genes was detected in mycotoxins-exposed (Figure 4A), common genes in both feeding groups (Figure 4B), and red yeast + mycotoxins (Figure 4C) compared to the control diet.

The results showed a high level of upregulation of a tumor suppressor gene *FBXO47* (Zhang et al., 2020) in hens fed with mycotoxins. A similar trend of upregulation was observed for *DUSP8* in the same feeding group. In addition, upregulation of a fatty acid biosynthesis gene *ELOVL2* was detected in the mycotoxins-exposed group. By contrast, *XDH,* a gene involved in the oxidative metabolism of purines which also functions as an important regulator in inflammatory cascades (Chen et al., 2017), was downregulated in MT vs. CON group. Similarly, downregulation of genes involved in the phase II detoxification process, *GSTA3* and *MGST1*, and an antioxidant gene *SOD1*, was detected in the same comparison group (Figure 4A).

In the common genes expressed in both mycotoxins-exposed and red yeast + mycotoxins groups, two genes involved in phase I (*CYP1A1* and *CYP1A2*) and phase II (*GSTA2*) of the detoxification process of xenobiotics, were downregulated (Figure 4B).

In the RY + MT feeding group, only two genes were upregulated compared with the CON group, namely a stress response gene *ATF4* and the gene *CDT1*, which regulates DNA replication initiation. In contrast, a tumorigenesis gene (*NFATC2*), a key cholesterol homeostasis gene (*ABCA1*), and a lipid metabolism gene (*NR1D2*), were downregulated when red yeast is included into the diet in this experimental group. Notably, downregulation of two genes contributing to xenobiotic metabolism, *CYP2C23a* and *CYP2C23b*, was detected in the red yeast feeding group compared to the control animals (Figure 4C).

### Functional annotation of differentially expressed genes in mycotoxins-exposed and red yeast + mycotoxins groups

**Figure 5:**
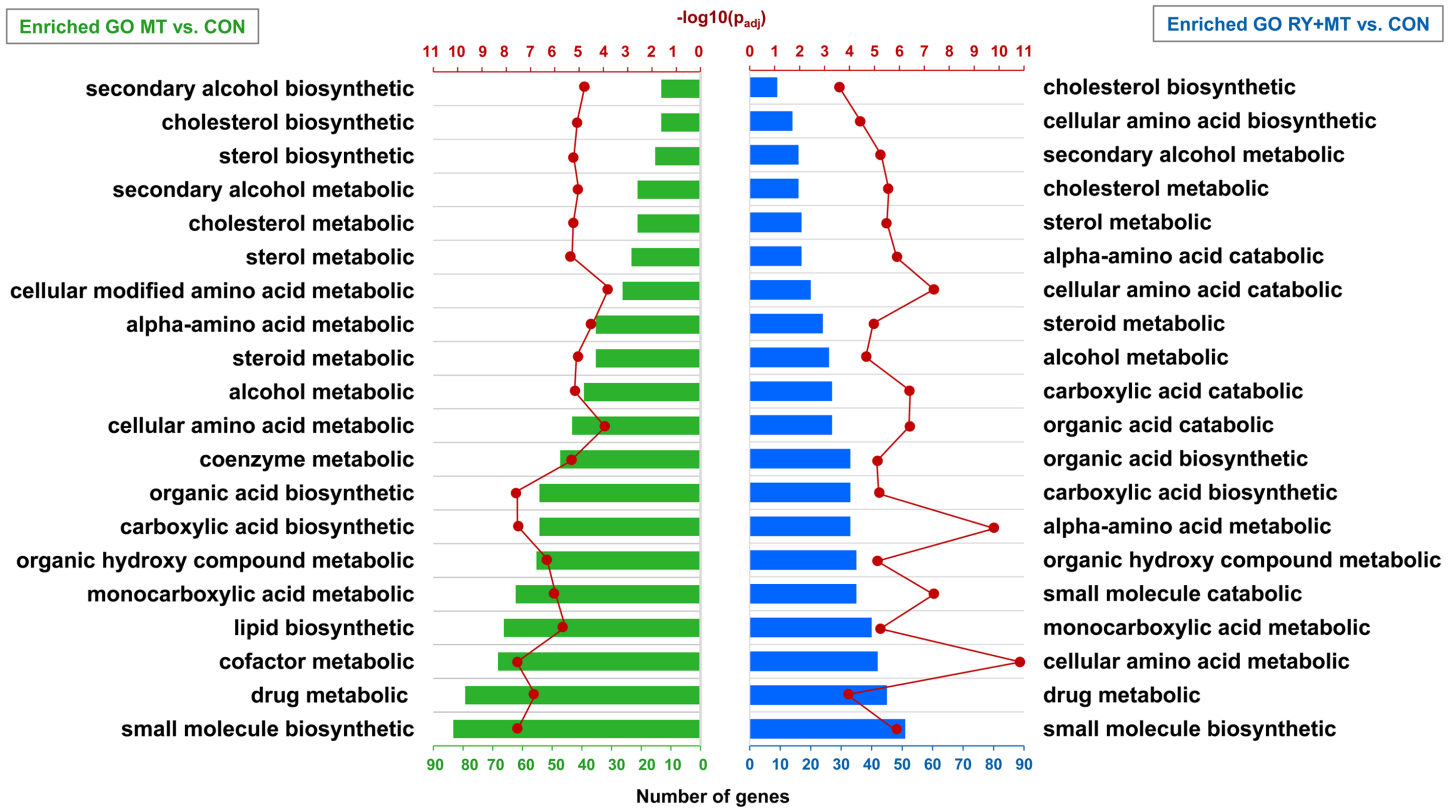
Significantly enriched terms in gene ontology (GO) analysis. The bar charts illustrate the annotation of GO categories in biological process in mycotoxins versus control (MT vs. CON) and in red yeast + mycotoxins versus control (RY + MT vs. CON). The vertical axis represents the top 20 significant enriched GOs (*p*_adj_ < 0.05) and the horizontal axis represents the number of genes in each GO term. The second horizontal axis indicates the significant level of each GO term -log_10_ (*p*_adj_ < 0.05).

The DEGs in the mycotoxins-exposed and red yeast + mycotoxins groups compared to the control group were subjected to functional enrichment analysis. The results of this analysis represent the biological function of the identified differentially expressed genes in the pathway and gene ontology (GO) categories. The top 20 significant identified GO terms (*p*_adj_ < 0.05) enriched in the biological process in the MT vs. CON and MT + RY vs. CON are shown in Figure 5 (*p*_adj_ < 0.05). The complete list of enriched GOs are provided in Supplementary Table 2, S2 and S3. The results revealed that the majority of enriched GOs in both MT and RY + MT diets compared to control diet play functional roles in metabolic process of drug, lipid, and amino acids such as drug metabolic, cholesterol metabolic, cellular amino acid metabolic, and alpha-amino acid metabolic. The difference between the identical enriched GOs in both experimental groups is reflected in the number of DEGs involved in each GO term and the level of significance in each experimental group. The unique enriched GOs in MT vs. CON were cofactor metabolic, lipid biosynthetic, coenzyme metabolic, cellular modified amino acid metabolic, sterol biosynthetic, and secondary alcohol biosynthetic. However, in the RY + MT vs. CON group, the unique identified GOs were small molecule catabolic, organic acid catabolic, carboxylic acid catabolic, cellular amino acid catabolic, alpha-amino acid catabolic, and cellular amino acid biosynthetic.

**Figure 6:**
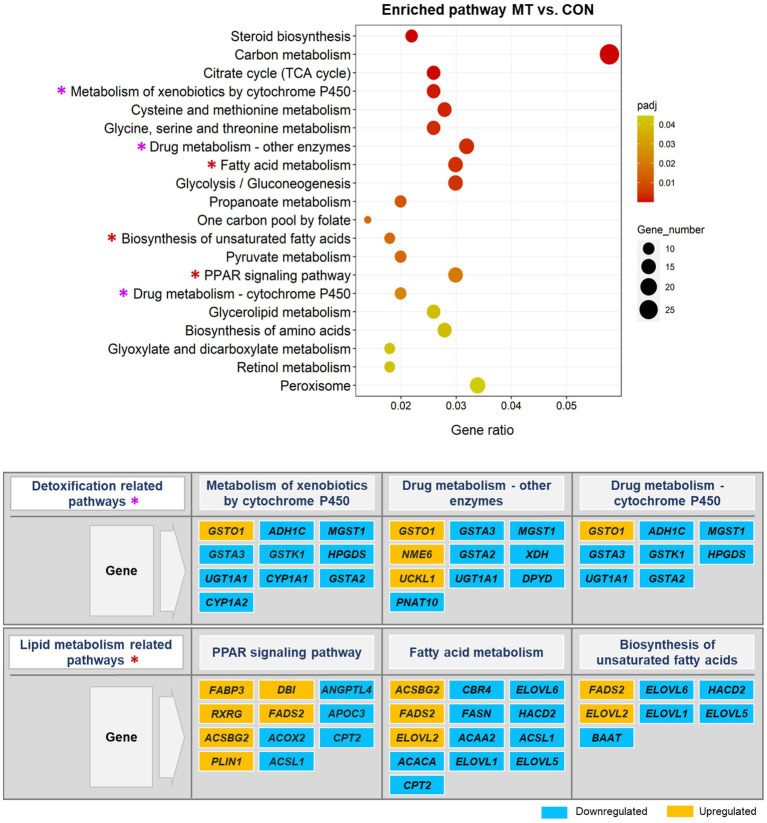
Significantly enriched pathways in the KEGG pathway analysis. The scatter plot illustrates the significantly enriched pathways (*p*_adj_ < 0.05) in mycotoxins versus control (MT vs. CON) group. The vertical axis represents the enriched pathway categories and the horizontal axis represents the gene ratio (the ratio of differentially expressed genes enriched in each pathway to the total number of genes in the pathway). The size and colour of dots indicate gene number and the range of *p*_adj_-value, respectively. The enriched detoxification and lipid metabolism pathways are marked with an asterisk (*) in the scatter plot and their significantly up- and down-regulated genes are represented in the figure.

**Figure 7:**
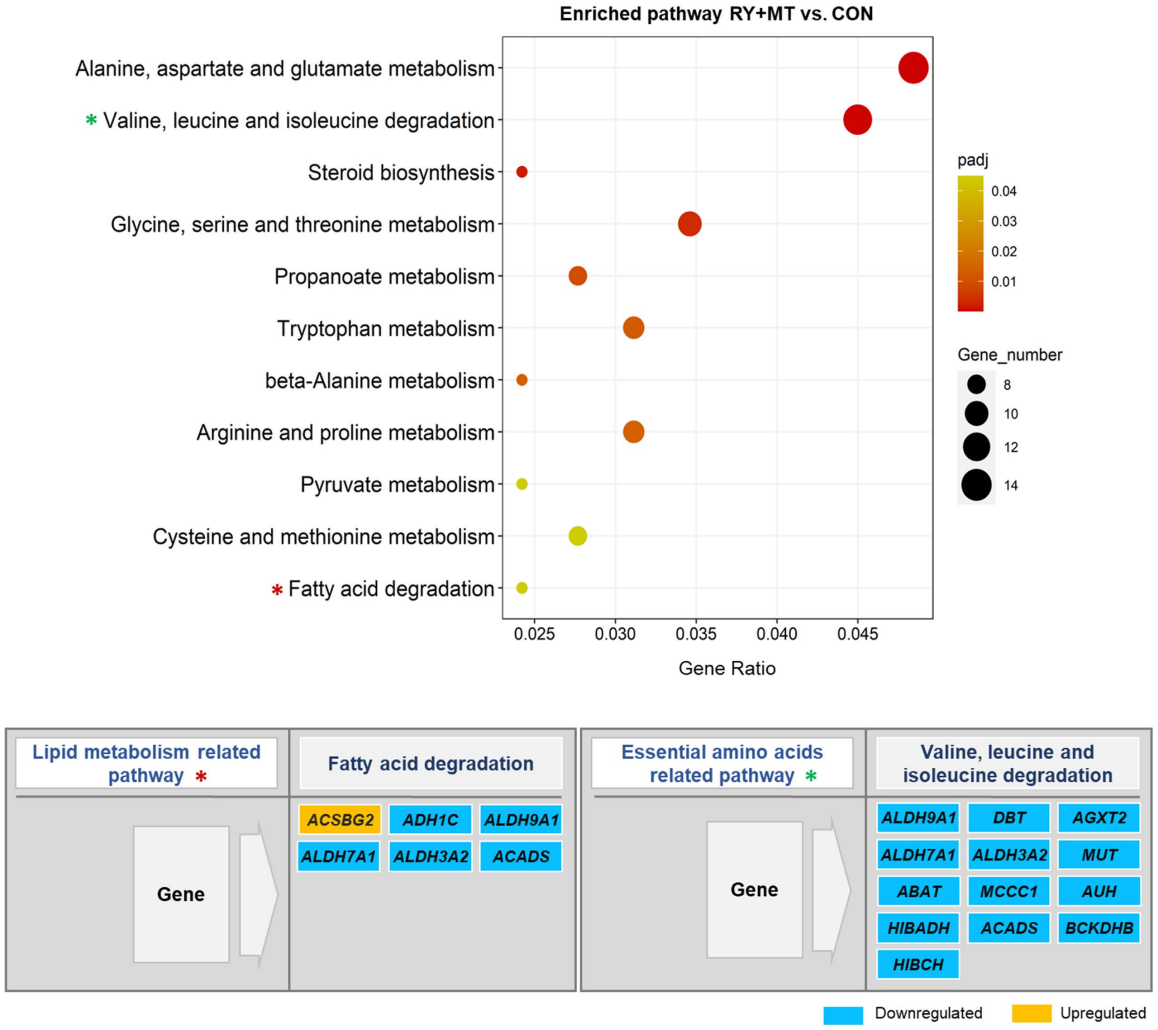
Significantly enriched pathways in the KEGG pathway analysis. The scatter plot illustrates the significantly enriched pathways (*p*_adj_ < 0.05) in red yeast + mycotoxins versus control (RY + MT vs. CON) group. The vertical axis represents the enriched pathway categories and the horizontal axis represents the gene ratio (the ratio of differentially expressed genes enriched in each pathway to the total number of genes in the pathway). The size and colour of dots indicate gene number and the range of *p*_adj_-value, respectively. The enriched lipid metabolism and essential amino acids related pathways are marked with an asterisk (*) in the scatter plot and their significantly up-and down-regulated genes are represented in the figure.

The pathways altered significantly (*p*_adj_ < 0.05) by DEGs in this study are shown in Figures 6, 7. Most of the pathways affected by mycotoxins contaminated diet compared to the control diet (MT vs. CON) were the major important metabolic pathways involved in detoxification processes (Figure 6). Among them, the significantly enriched metabolic pathways of principal interest in the mycotoxins-exposed group included the metabolism of xenobiotics by cytochrome 450, drug metabolism-other enzymes, and drug metabolism-cytochrome 450. In these pathways, some genes including an oxidative stress response gene (*GSTO1*), a metastasis suppressor gene (*NME6*), and a gene involved in tumor cell survival (*UCKL1*), were upregulated. However, most genes in these metabolic pathways, particularly genes involved in phase I and phase II detoxification mechanisms (e.g., *CYP1A2, GSTA2, GSTA3, MGST1*) were downregulated. Remarkably, the significant enrichment of the lipid metabolism-related pathways, namely PPAR signaling pathway, fatty acid metabolism, and biosynthesis of unsaturated fatty acids were detected in this comparison group in response to the increased mycotoxin-induced toxicity. In these pathways, many genes involved in fatty acid metabolism and biosynthesis (e.g., *FABP3, FADS2, ELOVL2*) were upregulated, however some of them (e.g., *ELOVL5, ELOVL6, HACD2*) were downregulated.

By contrast, none of the key detoxification and lipid metabolism pathways in the mycotoxins-exposed group were enriched in the diet supplemented with red yeast compared to the control group (RY + MT vs. CON, Figure 7). Instead, red yeast supplementation leads to downregulation of lipid metabolism and essential amino acids degradation related pathways in RY + MT vs. CON group. In this regard, upregulation of only one fatty acids synthesis gene (*ACSBG2*) and downregulation of several genes from the aldehyde dehydrogenase (ALDH) superfamily, encoding enzymes for detoxification of pharmaceuticals and environmental pollutants, was detected in the fatty acid degradation pathway in response to the inclusion of red yeast in the diet (Figure 7). Furthermore, all DEGs in the valine, leucine, and isoleucine degradation pathway, a pathway related to essential amino acid degradation, were downregulated in the RY + MT vs. CON group, suggesting the positive effect of red yeast supplementation in laying hen diet to improve animal health and protect from mycotoxin toxicity.

## Discussion

In the present study, transcriptome analysis of laying hen livers subjected to different feeding regimes revealed differences in gene expression profiles of hens fed with different diets compared to the control diet. Differential expression of a large number of genes was detected in response to long-term mycotoxin contaminated feed exposure, whereas the number of DEGs was significantly reduced when red yeast was included in the diet. Consideration of DEGs revealed the upregulation of some genes involved in apoptosis and tumor suppressor in experimental group fed with mycotoxins, while the expression of none of them was detected in feeding regimes containing red yeast. In contrast, gene involved in cellular detoxification process and antioxidant defense were downregulated in the mycotoxins-exposed group.

The long-term mycotoxin exposure leads to tissue damage as a consequence of cellular oxidative stress, resulting from the increased formation of free radicals (Zhang et al., 2014), which in turn can cause peroxidative damage in vital organs (Kumari and Singh, 2021). Lipid peroxidation is an oxidative attack, resulting from accumulation of reactive intermediates that affects cell membranes and induces apoptosis or necrosis programmed cell death (Ayala et al., 2014). Oxidative stress and the resultant increased lipid peroxidation are well-known effects of mycotoxicity reported in several studies in laying hens (Bócsai et al., 2015; Erdélyi et al., 2018; Kulcsár et al., 2021, 2023). To counteract lipid peroxidation, the cellular antioxidant defense system and its encoding genes are activated, whose activation is related to the dose of toxins and the duration of exposure (Mavrommatis et al., 2021). It should also be noted that the nature and extent of the detoxification process are highly specific to the toxic compounds and may vary between individuals depending on their genetic makeup (Mróz et al., 2022).

Expression of *CYP* family genes, which usually enhance the functionality of xenobiotic molecules in phase I detoxification (Mróz et al., 2022), can be induced or repressed in response to a variety of chemical and pathophysiological signals (Riddick et al., 2003, 2004). Increased transcription rate of *CYP* genes such as *CYP1A1* and *CYP1A2* and several other members of this family are known to encode metabolizing enzymes when exposed to endogenous and exogenous stimuli (Riddick et al., 2004). However, suppression of their expression has been investigated as a pathophysiological response to stress signals due to infectious or inflammatory stimuli (Riddick et al., 2004). Inflammation decreases *CYP* activity and downregulates its transcription and expression in the liver (Stanke-Labesque et al., 2020). Consistent with previous observations, the transcriptional expression of *CYP1A1* and *CYP1A2* genes was downregulated in the mycotoxins contaminated feeding group in this study, reflecting that a chronic inflammatory may damage the liver tissue in response to prolonged mycotoxin exposure. Downregulation of *IL1R1* and *XDH*, genes that respond to immunity and inflammation, in the same comparison group provides further evidence for this assumption. Likewise, the expression of genes involved in the phase II detoxification process, including *GSTA2, GSTA3*, and *MGST1,* was downregulated in the mycotoxins-exposed group in this study. A similar trend of downregulation was observed for genes with antioxidant activity such as *SOD1* in this feeding group. These results suggest that long-term mycotoxin exposure may leads to increased lipid peroxidation, which in turn can result in suppression of detoxification mechanisms and consequent tissue damage. Thus, it appears that the liver tissue of laying hens in the present study undergoes cell death programmed by apoptosis or necrosis, as upregulation of an apoptosis gene *DUSP8* and two tumor suppressor genes *KIAA1324* and *FBXO47* was detected in the mycotoxins contaminated feeding group. However, validation of these observations requires further research, which should focus on the physiological mechanism underlying lipid peroxidation and oxidative stress. In this regard, previous studies on long-term exposure to AFB1 indicated lipid deposition and increased hepatocyte apoptosis and histopathological changes in the liver of laying hens (Mughal et al., 2017; Liu et al., 2020). Similarly, prolonged exposure to AFB1 (Yarru et al., 2009) and OTA toxin (Kövesi et al., 2019) in broiler chickens suppressed detoxification mechanisms, and enhanced lipid peroxidation in the liver. The effect of short-term exposure to the single mycotoxin AFB1 (Erdélyi et al., 2018) and T-2 (Bócsai et al., 2015) in the liver of laying hens showed mild oxidative stress and initiated lipid oxidation, which was effectively eliminated by activation of the antioxidant defense system. Exposure to multiple mycotoxins simultaneously (DON, T-2, FB1) for a short time window in laying hens similarly showed activation of antioxidant defense in response to free radicals formation and inhibition of further lipid peroxidation, resulting in mild oxidative stress in the liver (Kulcsár et al., 2021, 2023). However, the effect of multiple mycotoxins in long-term exposure at the cellular level of vital organs in laying hens have not yet been fully described. Therefore, this study provides new insights into the synergistic effects of long-term exposure to combined mycotoxins in the liver of laying hens.

Red yeast as a novel biological binder of mycotoxins applied in this study altered the physiological and genetic response of hens to mycotoxicity. In the experimental group fed with MT + RY, the expression of genes encoding both phase I detoxification enzymes (*CYP1A1*, *CYP1A2*, *CYP2C23a*, *CYP2C23b*) and phase II enzymes (*GSTA2*) was significantly downregulated compared to the control animals, suggesting that mycotoxins may have been adsorbed by the red yeast in the digestive tract and its deleterious effects eliminated in liver cells. In contrast to the mycotoxins-exposed group, neither genes involved in antioxidant defense nor tumor suppressor genes were significantly differentially expressed in hens fed with red yeast. Notably, the expression of genes involved in lipid metabolism and the apoptosis process (e.g., *NFATC2, NR1D2, ULK2*) was downregulated in MT + RY vs. CON group. However, only one stress response gene *ATF4* was upregulated, possibly due to mild oxidative stress in the liver. These observations suggest that red yeast reduced the adsorption of mycotoxins by the liver in hens fed with MT + RY and the applied mycotoxins could not increase lipid peroxidation due to the adsorbent properties of red yeast during the application period. The red yeast cell wall acts as a biological agent that binds to mycotoxin upon intake of the contaminated feed in the gastrointestinal tract to eliminate its toxic effect on vital organs (Kanmanee et al., 2022; Srinual et al., 2022). In addition to the adsorption and antioxidant properties of red yeast, several studies in chicken have verified its beneficial effects in improving animal health and productivity, acting as a prebiotic with high nutritional value (Tapingkae et al., 2018; Kanmanee et al., 2022; Srinual et al., 2022). However, to the best of our knowledge, there are no studies on the effects of red yeast in interaction with multiple mycotoxins supplementation in the chicken diet at molecular and cellular levels in the liver of laying hens. We are the first to report the genetic response to feeding red yeast as a feed additive combined with multiple mycotoxins in the liver of laying hens using transcriptome profiling.

The results above were further verified by enrichment analysis, in which the detoxification and lipid metabolism pathways were significantly enriched in the feeding group contaminated with mycotoxins. In accordance with our findings, enrichment of pathways related to fatty acid metabolism was identified in AFB1 induced toxicity in the liver of laying hens (Liu et al., 2020). By contrast, none of these pathways were identified in the group fed with red yeast-mycotoxins combined diet in this study. Instead, the fatty acid degradation and essential amino acid degradation pathways were detected in this dietary group, where the DEGs in these pathways were downregulated, indicating the positive effect of red yeast on preventing tissue damage and consequent protection of animal health.

## Conclusion

In summary, this study indicated expression changes in different gene clusters in response to long-term feeding of laying hens with multiple mycotoxins, which can lead to adverse health effects. The application of red yeast as a feed additive in the mycotoxins contaminated diet significantly reduced the deleterious effects of mycotoxins in the liver of laying hens. These results suggest that red yeast has the potential to be used as a sustainable and environmentally friendly mycotoxin binding agent in the poultry feed industry to reduce health concerns for animals and increase food safety for human consumers.

## Data availability statement

All data generated and analyzed in this study are included within the article and its Supplementary material. The raw sequencing datasets used and analyzed in this study are deposited in the NCBI Sequence Read Archive (SRA) online repository, accession number via BioProject: PRJNA986678. Further use of the datasets generated and analyzed in this study requires the consent of both corresponding authors (SH and KG).

## Ethics statement

The animal study was approved by Maejo University Animal Care and Use Committee (MACUC), Thailand. All experiments of this study were performed in accordance with the standard guidelines and regulations for the treatment and use of laboratory animals at Maejo University, Chiang Mai, Thailand, under the approved permit number: MJUAN2560/15.

## Author contributions

SH: Data curation, Formal analysis, Investigation, Methodology, Visualization, Writing – original draft. BB: Writing – review & editing. SW: Project administration, Writing – review & editing. CK: Project administration, Writing – review & editing. OS: Project administration, Writing – review & editing. WT: Conceptualization, Funding acquisition, Writing – review & editing. KG: Conceptualization, Funding acquisition, Supervision, Writing – review & editing.

## Funding

This research was funded by National Research Council of Thailand (21551) and Office of National Higher Education Science Research and Innovation Policy Council by Program Management Unit Competitiveness (PMUC), Thailand (C10F630200). This research was partially supported by Chiang Mai University, Thailand.

## Conflict of interest

The authors declare that the research was conducted in the absence of any commercial or financial relationships that could be construed as a potential conflict of interest.

## Publisher’s note

All claims expressed in this article are solely those of the authors and do not necessarily represent those of their affiliated organizations, or those of the publisher, the editors and the reviewers. Any product that may be evaluated in this article, or claim that may be made by its manufacturer, is not guaranteed or endorsed by the publisher.
